# CSGBBNet: An Explainable Deep Learning Framework for COVID-19 Detection

**DOI:** 10.3390/diagnostics11091712

**Published:** 2021-09-18

**Authors:** Xu-Jing Yao, Zi-Quan Zhu, Shui-Hua Wang, Yu-Dong Zhang

**Affiliations:** 1 School of Computing and Mathematical Sciences, University of Leicester, Leicester LE1 7RH, UK; xy147@leicester.ac.uk; 2 Department of Civil and Coastal Engineering, University of Florida, Gainesville, FL 32608, USA; zhu.ziquan@ufl.edu

**Keywords:** COVID-19, machine learning, deep learning, convolutional neural network, Bayesian Optimization, chest CT

## Abstract

The COVID-19 virus has swept the world and brought great impact to various fields, gaining wide attention from all walks of life since the end of 2019. At present, although the global epidemic situation is leveling off and vaccine doses have been administered in a large amount, confirmed cases are still emerging around the world. To make up for the missed diagnosis caused by the uncertainty of nucleic acid polymerase chain reaction (PCR) test, utilizing lung CT examination as a combined detection method to improve the diagnostic rate becomes a necessity. Our research considered the time-consuming and labor-intensive characteristics of the traditional CT analyzing process, and developed an efficient deep learning framework named CSGBBNet to solve the binary classification task of COVID-19 images based on a COVID-Seg model for image preprocessing and a GBBNet for classification. The five runs with random seed on the test set showed our novel framework can rapidly analyze CT scan images and give out effective results for assisting COVID-19 detection, with the mean accuracy of 98.49 ± 1.23%, the sensitivity of 99.00 ± 2.00%, the specificity of 97.95 ± 2.51%, the precision of 98.10 ± 2.61%, and the F1 score of 98.51 ± 1.22%. Moreover, our model CSGBBNet performs better when compared with seven previous state-of-the-art methods. In this research, the aim is to link together biomedical research and artificial intelligence and provide some insights into the field of COVID-19 detection.

## 1. Introduction

In February 2020, the World Health Organization (WHO) named the cause of the Coronavirus Disease 2019 (COVID-19) as SARS-CoV-2 [1], which is a novel coronavirus that firstly been found in the human body. This virus shares a structural similarity of 87.1% [2] to the SARS-related virus found in bats, and 79.5% [3] to the SARS virus, which means novel coronavirus and SARS coronavirus belong to the same family of viruses but are not the same.

**Figure 1:**
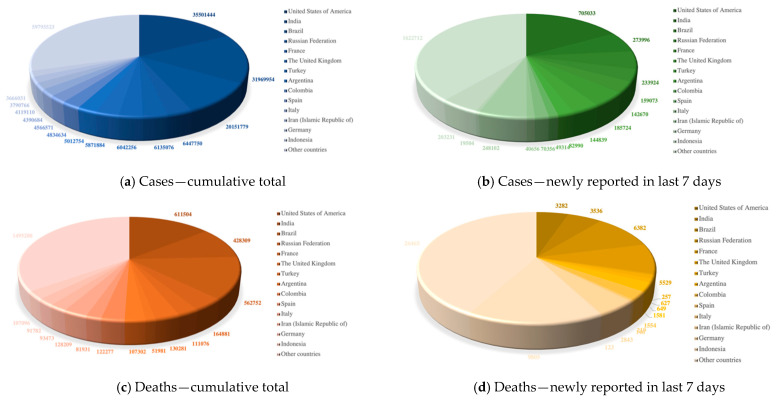
WHO Coronavirus (COVID-19) Dashboard.

According to the latest real-time statistics reported by the WHO in Figure 1, as of 10:37 a.m. Central European Summer Time (CEST) on 9 August 2021, there have been 202,296,216 confirmed cases of COVID-19 and 4,288,134 confirmed deaths over 225 countries, areas, or territories. Although vaccine doses have been administered in significant numbers, newly reported confirmed cases and deaths are still emerging every day.

From the current epidemiological investigation for COVID-19 [4], the patient of latent period and some confirmed patients has no difference in appearance with the normal person but still has infectivity. Therefore, it is urgent to expand the coverage of COVID-19 inspection, accelerate the speed of detection, improve the accuracy of detecting infected persons including the asymptomatic infected persons as early as possible to avoid secondary transmission [5]. However, at present, based on a variety of practical considerations, several countries around the world are trying to relax social restrictions, which poses great challenges to COVID-19 detection level, especially in places with high traffic density such as customs, airports, and stations. Thus, to quickly improve the COVID-19 diagnostic level, scientists and medical institutions worldwide are actively engaged in in-depth research into the COVID-19 diagnosis.

At present, the commonly used standard method of COVID-19 detection is the reverse transcription-polymerase chain reaction (RT-PCR) test. Nevertheless, RT-PCR usually takes up two days to complete and sometimes needs repetition or some other auxiliary detection approach in test to exclude potential false negatives [6]. Care also needs to be taken during testing to prevent environmental contamination of the samples. Therefore, to improve the efficiency of COVID-19 detection, many medical institutions consider chest computed tomography (CT) scan as an early diagnostic tool for suspected coronavirus infection. Chest CT is a common basic diagnostic technique for pneumonia, which uses X-ray CT to examine the chest [7]. Fang, Zhang [6] compared the sensitivity of chest CT and RT-PCR in COVID-19 detection. The results indicate that chest CT is an effective method for screening COVID-19 patients, especially those who are tested negative for RT-PCR.

However, manual labelling and diagnosing of CT images may be time-consuming and labour-intensive. Human experts may also misdiagnose due to some external interference or subjective factors, especially when the lesion degree is mild. Hence, using computer-aided technologies to assist expert diagnosis has become a good choice [8,9]. Deep learning technology has also far achieved many contributions in dealing with CT images. Onishi, Teramoto [10] proposed a novel deep learning system that combined a deep convolutional neural network and generative adversarial networks for pulmonary-nodule classification. Wang, Shen [11] presented a raw patch-based convolutional neural network for the detection of lung nodules. Peng, Kang [12] used a residual convolutional neural network for predicting the response of transarterial chemoembolization in hepatocellular carcinoma. As displayed in Table 1, three pieces of the latest research from Wang and Wong [13], Dey, Rajinikant [14], and Abbas, Abdelsamea [15] provide systematic reviews of deep learning techniques in detecting COVID-19. These all prove that deep learning methods can well approximate expert skills, and at the same time find novel relationships not already apparent to humans.

GoogLeNet is a classic deep learning structure proposed by Szegedy, Ioffe [16]. Our novel model was proposed based on transfer learning from GoogLeNet. In addition, to prevent gradient explosion or dispersion and keep most activation functions away from their saturated region, more batch normalization relevant blocks were added into the newly designed layers. In this way, the robustness of the model to different hyperparameters during training will be improved and the optimization process will become smoother. This smoothness makes the behavior of the gradient more predictable and stable, allowing for faster training [17].

When dealing with deep neural networks, the selection of hyperparameters is always a problem. Manual parameter tuning is time-consuming and may not achieve optimal results. The advantage of Bayesian Optimization (BO) in parameter tuning is sample efficiency. In other words, if we consider one step as training the neural network with a set of hyperparameters, BO can find a better choice of hyperparameters with very few steps. In addition, BO does not ask for the gradient, which is difficult to be obtained from the hyperparameter of the neural network under general circumstances. These two benefits make BO become the algorithm we chose to tune the neural network in this experiment.

In this study, we linked biomedical research and artificial intelligence together to propose a novel, rapid, stable deep learning framework called CSGBBNet with excellent accuracy for distinguishing between COVID-19 patients and healthy people. There are four main improvements in our study. The first is we proposed a novel image pre-processing model—COVID-Seg based on the Maximum entropy threshold segmentation method, which can accurately outline the lung area. The ‘COVID’ here stands for ‘COVID-19 detection’, and the ‘Seg’ signifies ‘Segmentation process’. The second is, in this COVID-Seg model, we introduced an ‘erosion and dilation’ based process to refine the output segmented image. This is the first time this combination of techniques being applied to the COVID-19 detection field, and the results show that this COVID-Seg model has a very significant effect on improving the model performance. Thirdly, we presented a novel GBBNet through restructuring, retraining the GoogLeNet model, and adding more batch normalization relevant blocks into it. The fourth is, in our GBBNet, we used Bayesian Optimization as the hyperparameter tuning algorithm to find a better choice of hyperparameters rapidly. In the name ‘GBBNet’, the ‘G’ represents ‘GoogLeNet’; the first ‘B’ stands for ‘Batch normalization relevant blocks’; the second ‘B’ stands for ‘Bayesian Optimization’. These four improvements can help enrich the performance of our model specially built for COVID-19 detection. Section 2, Section 3, Section 4 and Section 5 will introduce the dataset, methodology, experimental results, and conclusions of our study, respectively.

## 2. Dataset

**Figure 2:**
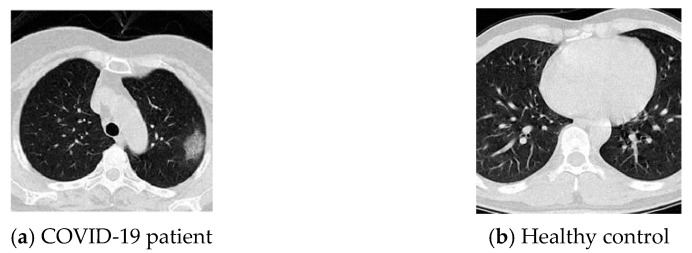
Illustration of the COVID-19 dataset ‘COVID Academic’.

The dataset named ‘COVID Academic’ is from [18]. It consists of 196 slice images and have been carefully labelled by human experts from the Fourth People’s Hospital of Huai’an City. It can be divided into two parts: one part of 98 CT scan images from patients with novel coronavirus pneumonia and the other part of 98 CT scan images from healthy control (HC). The example images are displayed in Figure 2. The instrument and settings for the CT acquisition are Philips Ingenuity 64 row spiral CT machine, Mas: 240, KV: 120, layer spacing 3 mm, layer thickness 3mm, Mediastinum window (Width, Level: 350, 60 in Hounsfield units), thin layer reconstruction according to the lesion display. The slice images using the agreed slice level selection (SLS) method: (i) selecting the slice with the largest size and number of lesions for the patients with COVID-19 and (ii) for the normal cases, any level of the slice can be chosen. Through utilizing this SLS method, 196 slice images were extracted with a resolution of 1024 × 1024 from COVID-19 patients and healthy people.

## 3. Methodology

The abbreviation list and symbol list for this section are shown in Table 2 and Table 3.

### 3.1. Preprocessing

**Figure 3:**
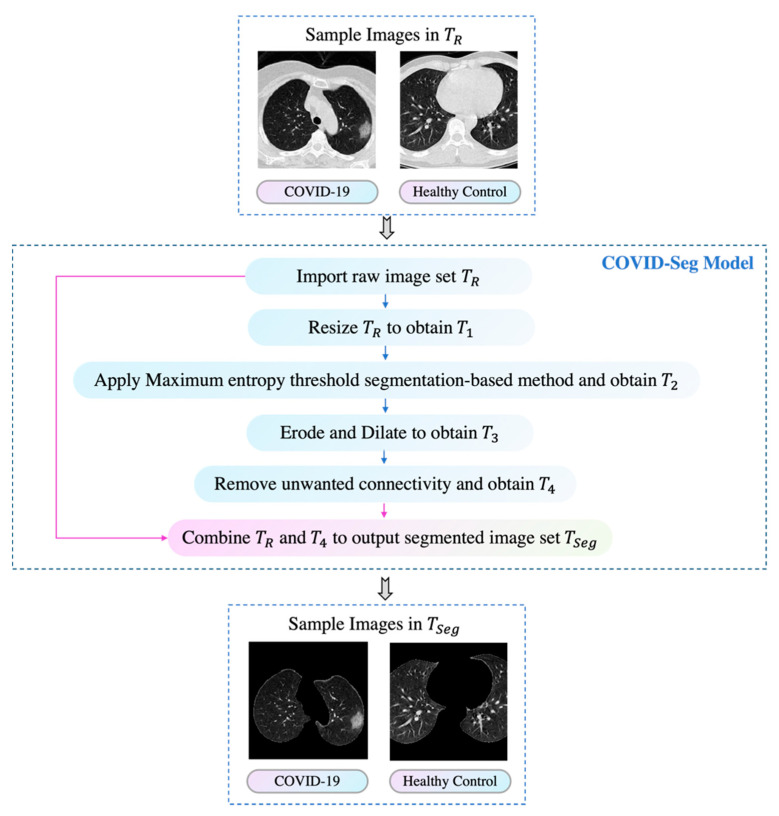
General process of COVID-Seg model.

Preprocessing has shown many benefits in medical image analysis [9,19]. We presented a novel model—COVID-Seg for segmenting lung regions in each CT image. Tissues regions other than the lung regions, such as the regions of heart, ribs, and thoracic vertebrae, were removed because no changes in the CT image will appear if these regions were infected by the COVID-19 virus. Taking the Figure 2 images as input examples, the general process of the COVID-Seg model is illustrated in Figure 3.

In this model, we first utilized the maximum entropy threshold segmentation method to obtain the preliminary mask of lung regions by classifying each pixel in the image [20,21], which will be discussed later in Section 3.1.1. After that, we refined the mask via some appropriate image processing techniques such as ‘erosion and dilation-based process’, which will be discussed later in Section 3.1.2.

In this study, the COVID-Seg model can help remove the background interference factors such as the regions of the heart, ribs, and thoracic vertebrae, and thus improves the classification performance [22]. The basic steps for the COVID-Seg model are as follows:

Step 1: Import the raw image set and store them into variable $T_{R}$.

Step 2: Resize the images to the same size. Since the neural network receives inputs of the same size [23], all images need to be resized to a fixed size before inputting them into the neural network. 224 × 224 is a very suitable choice for the pre-trained model in our research.

(1) $$T_{1}=F_{resize}\left(T_{R}\right)$$

where $F_{resize}$ refers to a function of resizing the images to the same size.

Step 3: Use the Maximum entropy threshold segmentation-based method to obtain the preliminary mask set.

(2) $$T_{2}=F_{mets}\left(T_{1}\right)$$

where $F_{mets}$ refers to a function of utilizing the Maximum entropy threshold segmentation-based method.

Step 4: To refine the preliminary mask, we set the different sizes of erosion and dilation squares and apply ‘erosion and dilation-based process’ to the mask.

(3) $$T_{3}=F_{ed}\left(T_{2}\right)$$

where $F_{ed}$ refers to a function of ‘erosion and dilation-based process’.

Step 5: Remove unwanted connectivity areas of the mask and then make an inversion of the values in the mask (change all the ‘0’ into ‘1’ and all the ‘1’ into ‘0’) to obtain the final refined mask set. This inversion needs to be taken for letting the areas we are interested in and want to retain in the mask be set to ‘1’ and the areas we want to remove in the mask be set to ‘0’.

(4) $$T_{4}=F_{crwc}\left(T_{3}\right)$$

where $F_{crwc}$ refers to a function of choosing and retaining the wanted connectivity areas.

Step 6: Conduct element-wise multiplication between the final refined mask set and the original image set to output the segmented image set.

(5) $$T_{Seg}=F_{comb}\left(T_{4},T_{R}\right)$$

where $F_{comb}$ refers to a function of conducting an element-wise multiplication process (.*) between the final refined mask and the original image to get the final segmented image.

For easier understanding, the pseudocode of our proposed COVID-Seg model is listed in Algorithm 1.
**Algorithm 1** Pseudocode of our proposed COVID-Seg model**Input:** Original Image set $T_{R}$**Phase I: Resize** Resize the images to 224 × 224: $T_{1}=F_{resize}\left(T_{R}\right)$;**Phase II: Apply Segmentation**Apply Maximum entropy threshold segmentation-based method to obtain the preliminary mask set: $T_{2}=F_{mets}\left(T_{1}\right)$;**Phase III: Refine**Erode and dilate the mask: $T_{3}=F_{ed}\left(T_{2}\right)$;
Remove unwanted connectivity areas of the mask and make an inversion of the values in the mask to obtain the final refined mask set: $T_{4}=F_{crwc}\left(T_{3}\right)$;**Phase IV: Combine**Combine the final refined mask set and the original image set together to obtain the segmented image set: $T_{Seg}=F_{com}\left(T_{4},T_{R}\right)$.**Output**: The segmented image set $T_{Seg}$

#### 3.1.1. Improvement 1: Using Maximum Entropy Threshold Segmentation-Based Method to Get the Preliminary Mask

**Figure 4:**
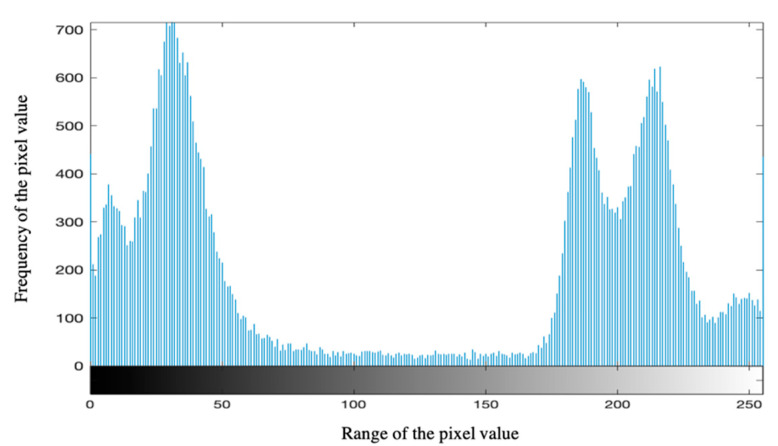
An illustration of histogram graph for a COVID-19 patient CT scan image.

Maximum entropy threshold segmentation [21] was utilized to help classify each pixel in the given image and remove the background interference factors, and thus improves the classification performance. An illustration of the histogram graph for Figure 1a is shown in Figure 4.

Assuming an image *I* contains *N* pixels, the probability p(i) of the occurrence of the pixel value equals *i* in the image can be defined as:

(6) $$p\left(i\right)=\frac{h\left(i\right)}{N}\;\;\;\;\;s.t.\;0\leq i<k\;\text{,}\sum_{i=0}^{k-1}h\left(i\right)=N$$

where *h*(*i*) is the y-axis value in Figure 4 and refers to the frequency of the pixel value equals *i* in the image; *i* stands for the x-axis value in Figure 4 and typically takes integer values from 0 to 255; *k* stands for the limited range of pixel value (e.g., 256).

The cumulative distribution function *P*() can be defined as:

(7) $$P\left(i\right)=\frac{H\left(i\right)}{H\left(k-1\right)}=\frac{H\left(i\right)}{N}=\overset{i}{\underset{j=0}{\sum }}\frac{h\left(j\right)}{N}=\overset{i}{\underset{j=0}{\sum }}p\left(j\right)\;\;\;\;\;s.t.0\leq i<k$$

where *H* stands for the cumulative histogram corresponding to the cumulative probability.

The probability of the occurrence of each gray level *x* (with the abscissa value of *I* as *u*, and the ordinate value of *I* as *v*) can be expressed as:

(8) p(x)=p(I(u,v)=x)

Because all probabilities need to be known in advance, these probabilities are also called prior probabilities which can be estimated by observing the frequency of the corresponding gray value in one image or more images. The probability vectors for *k* different gray values, *x* = 0, …, *k* − 1 can be expressed as: p(0), p(1),… , p(k−1). According to Equation (6), the probability distribution *p*(*x*) of the image is:

(9) $$p\left(x\right)=\frac{h\left(x\right)}{N}\;\;\;\;\;s.t.0\leq x<k,\;0<p\left(x\right)<1$$

According to Equation (7), the corresponding cumulative probability distribution function is:

(10) $$P\left(x\right)=\overset{x}{\underset{i=0}{\sum }}\frac{h\left(x\right)}{N}=\overset{x}{\underset{i=0}{\sum }}p\left(i\right)\;\;\;s.t.\;P\left(0\right)=p\left(0\right),P\left(k-1\right)=1$$

Given an estimated probability density function *p*(*x*) in the digital image, the entropy in the digital image can be defined as:

(11) $$H\left(I\right)=\underset{u,v}{\sum }p\left(I\left(u,v\right)\right)\cdot \log_{b}\left\{\frac{1}{p\left(I\left(u,v\right)\right)}\right\}=-\underset{u,v}{\sum }p\left(x\right)\cdot \log_{b}\left(p\left(x\right)\right)$$

Given a specific threshold *y* (0 ≤ *y* < *k* − 1), for the two image regions $Y_{0}$ and $Y_{1}$ segmented by this threshold, the estimated probability density function can be expressed as:

(12) $$Y_{0}:\left(\frac{p\left(0\right)}{P_{0}\left(y\right)}\;\frac{p\left(1\right)}{P_{0}\left(y\right)}\ldots \;\frac{p\left(y\right)}{P_{0}\left(y\right)}\;0\;0\ldots 0\right),Y_{1}:(0\;0\ldots 0\;\frac{p\left(y+1\right)}{P_{1}\left(y\right)}\;\frac{p\left(y+2\right)}{P_{1}\left(y\right)}\ldots \;\frac{p\left(k-1\right)}{P_{1}\left(y\right)})$$

(13) $$P_{0}\left(y\right)=\overset{y}{\underset{i=0}{\sum }}p\left(i\right)=P\left(y\right),\;P_{1}\left(y\right)=\overset{k-1}{\underset{i=y+1}{\sum }}p\left(i\right)=1-P\left(y\right)$$

where $P_{0}\left(y\right)$ and $P_{1}\left(y\right)$ respectively represent the cumulative probability of background and foreground pixels segmented by *y* threshold, and the sum of them is 1.

The corresponding entropy of background and foreground are shown as follows:

(14) $$H_{0}\left(y\right)=-\overset{y}{\underset{j=0}{\sum }}\frac{p\left(i\right)}{P_{0}\left(y\right)}\log\left(\frac{p\left(i\right)}{P_{0}\left(y\right)}\right)\;,H_{1}\left(y\right)=-\overset{k-1}{\underset{i=y-1}{\sum }}\frac{p\left(i\right)}{P_{1}\left(y\right)}\log\left(\frac{p\left(i\right)}{P_{1}\left(y\right)}\right)$$

Under this threshold, the total entropy of the image is:

(15) $$H\left(y\right)=H_{0}\left(y\right)+H_{1}\left(y\right)$$

Subsequently, we calculated the total entropy of the image under all the segmentation thresholds and then found out the maximum entropy. The final threshold should be the segmentation threshold corresponding to the maximum entropy. The pixel in the image whose gray value is larger than this threshold would act as the foreground, and the pixel whose gray value is smaller than the threshold would act as the background [24].

#### 3.1.2. Improvement 2: Using Erosion and Dilation-Based Technique to Refine the Mask

However, in the mask set produced by entropy threshold segmentation, there is a severe problem that some lesion regions that contain useful information have been eliminated together with the heart, ribs, and thoracic vertebrae. Hence, to refine the mask, we utilized a method based on the Erosion and Dilation processing technique, which is a collection of nonlinear operations related to the morphology of objects in an image. This method relies only on the relative ordering, rather than the numerical values of pixel values, and thus is especially suited to the processing of binary images—the mask images [25,26]. Through utilizing this technique, we probed each mask by positioning a small structuring element at all possible locations in the mask, then compared that element with the corresponding neighborhood of pixels, and finally conducted the refining process.

Erosion with small (e.g., 2 × 2–5 × 5) square structuring elements can shrink the object by stripping away a layer of pixels from both the inner and outer boundaries of regions. Through utilizing erosion, we can let the holes and gaps between different regions become larger to eliminate some useless small details in the mask. While dilation has the opposite effect to erosion—it adds a layer of pixels to both the inner and outer boundaries of regions. Through utilizing dilation, we can make the holes enclosed by a single region, the gaps between different regions become smaller, and let the small intrusions into boundaries of a region be filled in [27,28]. Results of dilation or erosion are influenced by both the size and iterated utilization times of a structuring element: A larger structuring element and more times of utilization would bring a more pronounced effect. Furthermore, the result of erosion or dilation with a larger structuring element will be similar to the result obtained by iterated erosion or dilation using a smaller structuring element of the same shape. In other words, if *s*_1_ and *s*_2_ are a pair of structuring elements identical in shape, with *s*_2_ twice the size of *s*_1_, then

(16) $$f⊖s_{2}\approx \left(f⊖s_{1}\right)⊖s_{1}$$

where f⊖s denotes a new binary image produced from the erosion of a binary image *f* by a structuring element *s*.

We worked on adjusting the size and utilization times of the structuring element to achieve our desired refined mask. For ‘COVID Academic’, after many attempts, we found that the most suitable refining method is:

(17) $$r=\left(p⊖s_{1}\right)⊖s_{1}⊖s_{1}⊖s_{1}$$

where *r* represents the refined mask, *p* represents the preliminary mask.

Since the dilation and erosion are dual operations that have opposite effects, when we found the mask have been excessively eroded, we added the dilation step to recover the useful information in the image through recovering the mask:

(18) $$r=\left(p⊖s_{1}\right)⊖s_{1}⊖s_{1}⊖s_{1}⊕s_{q}$$

where *q* is the size of square structuring element which would be decided according to different degrees of excessive erosion; f⊕s denotes a new binary image produced from the dilation of a binary image *f* by a structuring element *s*.

**Figure 5:**
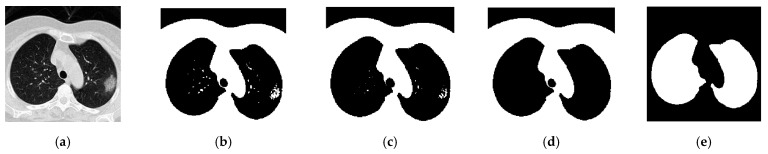
Sample significant output results during the process of using Erosion and Dilation-based technique to refine the mask for image lung regions: (**a**) Original Image. (**b**) Apply Maximum entropy threshold segmentation-based method to obtain the mask ($T_{2}$). (**c**) Erode and dilate the mask ($T_{3}$). (**d**) Remove unwanted connectivity areas of the mask. (**e**) Make an inversion of the values in the mask to achieve the final refined mask ($T_{4}$).

In the following steps, we repeatedly used the algorithm of selecting the largest connected areas and made an inversion of the values in the mask to retain the areas we are interested in. A series of sample significant output we got from the erosion and dilation-based process is as displayed in Figure 5.

### 3.2. Transfer Learning Model

Many neural networks increase the depth to achieve better training performance. However, deepen the network will meanwhile bring several negative effects, e.g., overfitting, gradient disappearance, and gradient explosion. Unlike them, GoogLeNet improves the training results by making more efficient usage of computing resources [29,30].

We restructured and retrained the GoogLeNet by replacing the layers after Inception Block 5b with our newly designed layers containing the Conv Block and incorporated the framework with Bayesian Optimization algorithm to propose a new network named GBBNet for the COVID-19 classification task.

**Figure 6:**
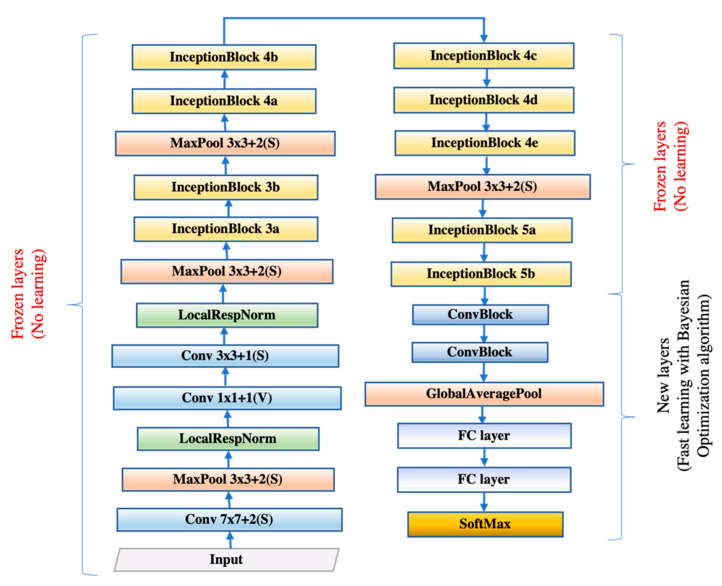
The structure of our proposed GBBNet.

The basic structure of GBBNet is illustrated in Figure 6. The numbers in the convolutional layer block and the max-pooling layer block represent the exact information of the layer. For instance, Conv 1 × 1 + 1 (V) represents a convolutional layer with 1 × 1 filter size, stride 1, and ‘valid’ padding; MaxPool 3 × 3 + 2 (S) represents a max-pooling layer with 3 × 3 filter size, stride 2, and ‘same’ padding.

**Figure 7:**
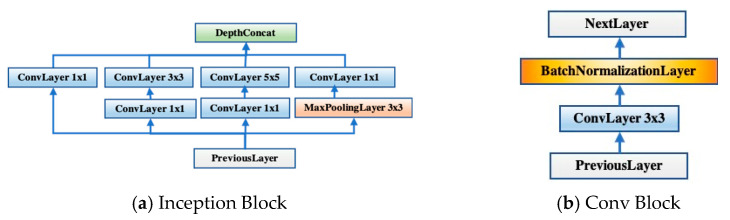
The basic structures of blocks used in GBBNet.

The Inception Block structure, which can be seen in Figure 7a, is reserved for two advantages. On the one hand, utilizing a 1 × 1 convolution block can raise and lower the dimension. On the other hand, convolution and reintegration on multiple scales can be conducted at the same time, which saves much time [16,31]. After the inception structure, two new Conv Blocks with batch normalization layers as illustrated in Figure 7b were added. The reason would be discussed in Section 3.2.1.

Global average pooling then undertook the process of feature map reduction to convert a feature map of w × w × c to 1 × 1 × c. Subsequently, the original fully connected layer (FCL) was altered to a new randomly initialized FCL of two neurons since the original one was used to conduct 1000 categories classification for ImageNet. And the classes in that 1000 categories are not associated with the main classification task for our research. The new classification layer also only has two classes. (COVID-19 patient and healthy people). Next, we utilized a precomputation strategy for retraining [32]. We firstly set the cognate learning factor of specified layers to zero for freezing those layers and then calculated the activation maps at the last frozen layer, ‘inception_5b-output’ for all the images. Finally, we saved the feature maps to local disk and used them as input for training the trainable layers.

#### 3.2.1. Improvement 3: Adding More Batch Normalization Relevant Blocks to Offset the Impact of Different Scales

In our research, if we tolerate the existence of covariate shift- the difference of characteristic distribution between different parts of the dataset, then the different distribution of input values would lead to the difference in input feature values. After multiplying the matrix with the weight, difference values with large deviation would be generated. Due to the reason that our deep learning network needs to be updated and improved through training, little changes in the difference value will have a deep impact on the back layer. The greater the deviation, the more obvious the impact will be. Therefore, to avoid these phenomena causing gradient divergence during backpropagation, we decided to add more batch normalization relevant blocks in the network to offset the impact of different scales and, at the same time improve the performance of our framework. BN can standardize the input values and reducing the scale differences to the same range, which brings two main benefits. On the one hand, this improves the convergence degree of gradient and speeds up the training speed. On the other hand, each layer can face the input value of the same characteristic distribution as far as possible, which reduces the uncertainty brought by changes and also reduces the influence on the back-end network, leading the network of each layer becomes relatively independent [33,34].

Assume BN is used to normalize the layer’s input $X=\left\{x_{1},\;x_{2},\;\ldots ,x_{n}\;\right\}$ to guarantee the output $Y=\left\{y_{1},\;y_{2},\;\ldots ,\;y_{n}\right\}$ to have a uniform distribution [35]. The mini-batch mean *µ* can be calculated as

(19) $$\mu_{X}=\frac{1}{n}\left(\overset{n}{\underset{i=1}{\sum }}x_{i}\right)$$

The mini-batch variance $\sigma^{2}$ can be calculated as

(20) $$\sigma_{X}^{2}=\frac{1}{n}\overset{n}{\underset{i=1}{\sum }}{\left(x_{i}-\mu_{X}\right)}^{2}$$

The input *x*_i_ ∈ *X* would be normalized to $\overset{ˇ}{x_{i}}$ according to

(21) $$\overset{ˇ}{x_{i}}=\frac{\left(x_{i}-\mu_{X}\right)}{\sqrt{\left(\sigma_{X}^{2}+\varphi \right)}}$$

where φ is a constant added to the mini-batch variance to improve numerical stability and is set to $10^{-4}$ in our experiment.

And the transformation for achieving *y*_i_ ∈ *Y* would be as

(22) $$y_{i}=\xi \overset{ˇ}{x_{i}}+\lambda$$

where ξ and λ are two parameters going to be learned during training.

Afterwards, the output $y_{i}$ would be sent to the next layer and the normalized $\overset{ˇ}{x_{i}}$ remains internal to the current layer.

#### 3.2.2. Improvement 4: Incorporating Bayesian Optimization in Network

Tuning parameters is an essential step that has a significant impact on model performance in machine learning. Traditional manual tuning is time-consuming and may not obtain optimal results especially when the network is complex and decided by many hyperparameters. The subsequent emerging tuning methods such as Grid Search and Random Search, though free of manpower, still take a long time and are prone to local optimal situations when dealing with non-convex functions [36,37,38,39].

**Figure 8:**
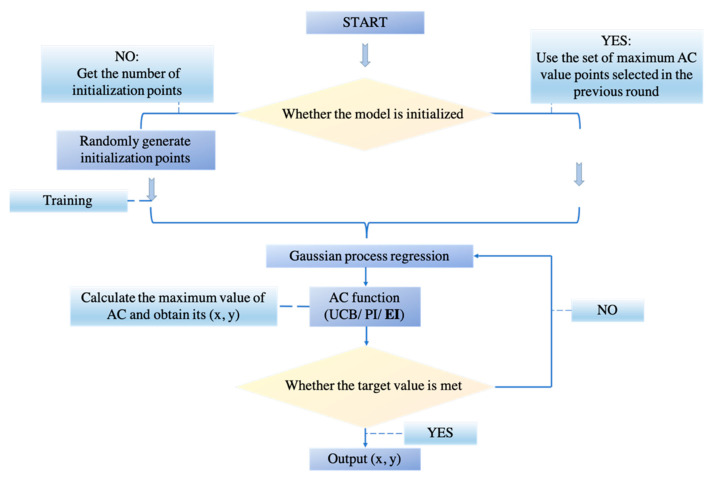
The pipeline of Bayesian Optimization.

In this research, we incorporated Bayesian Optimization for two main reasons. Firstly, it saves more time because they have fewer iterations. Secondly, it is also robust when dealing with non-convex problems. Figure 8 portrays the pipeline for Bayesian Optimization in our experiment.

In our experiment, the acquisition (AC) function we chose is the Expected Improvement (EI) function, as it solves the problem of PI by considering how much larger the unknown point is than the known maximum point. The equation [40] is as below:

(23) $$EI\left(x\right)=\{\begin{array}{cc} \left(\mu \left(x\right)-f\left(x^{+}\right)\right)\Phi \left(z\right)+\sigma \left(x\right)\phi \left(z\right) & \sigma >0\\ 0 & \sigma =0 \end{array}$$

(24) $$z=\frac{\mu \left(x\right)-f\left(x^{+}\right)}{\sigma }$$

where *x* is an observed point; μ is the mean of all the observation points; $f\left(x^{+}\right)$ represents the current maximum value; σ is the standard deviation of all observation points; Φ() and ϕ() denote the cumulative distribution function (CDF) and probability density function (PDF) of the standard normal distribution respectively.

Besides, we set the range of the following hyperparameters as displayed in Table 4 to fine-tune the model.

The best learning rate and learning drop factor would depend on their based dataset and the network. Momentum adds inertia to the parameter updates by having the current update contain a contribution proportional to the update in the previous iteration. L2Regulization is usually used to prevent the circumstance of overfitting. Thus, they are all good choices of variables for us to search the space for finding a suitable value.

### 3.3. K-Fold Cross-Validation

The main reason for adopting K-fold in this experiment is it performs well in processing small datasets. The original dataset needs to be grouped, with one part as a training set and one part as a testing set. In the traditional machine learning process, the testing set will not participate in the training process, which wastes some parts of the data. This limits the optimization of the model, especially when dealing with a small dataset because data determines the upper limit of program performance [41,42,43,44].

**Figure 9:**
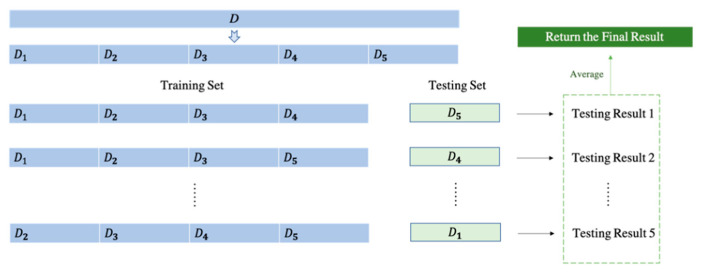
An illustration of fivefold cross-validation.

Thus, it is significant to make good use of data sets, especially for this experiment. K-fold cross-validation will randomly divide the training data into *K* folds, do *K* times of training, and get *K* testing results, which reports unbiased performances. In this way, all data sets can be well utilized, and finally, the model performance can be expressed reasonably utilizing averaging [45]. Considering the factor of overfitting and running time, we intended to choose five as the *K* value for our experiment. An illustration of fivefold cross-validation is shown in Figure 9. The *D* in the figure represents ‘Dataset’. 

### 3.4. Implementation of Our Proposed CSGBBNet

We proposed the framework of CSGBBNet by combining the COVID-Seg model and the GBBNet. The basic steps for implementation are as followed.

Step 1: Import the original Image set $T_{R}$ and its ground truth label $G_{t}$. Conduct the image pre-processing using COVID-Seg.

Step 2: Load the PTN model VGG16 [40], VGG19 [40], and GoogLeNet, and then store them into variables $M_{VGG16}$, $M_{vgg19}$ and $M_{GoogLeNet}$, respectively.

Step 3: Pre-train each model and make a comparison on their testing accuracy results to select the optimum one with maximum accuracy and then store the model into variable $M_{0}$. Suppose $L_{0}$ as its number of learnable layers.

Step 4: Remove the last *L* learnable layers from $M_{0}$ to get a new $M_{1}$,

(25) $$M_{1}=F_{rl}\left(M_{0},\;L\right)$$

where $F_{rl}$ refers to a function of removing layers, and *L* refers to the number of specific last layers to be removed.

Step 5: Add five new designed layers/blocks into $M_{1}$ to get a new $M_{2}$,

(26) $$M_{2}=F_{andl}\left(M_{1},5\right)$$

where $F_{andl}$ refers to a function of adding newly designed layers, and the constant 5 refers to the number of newly designed layers/blocks that would be appended to $M_{1}$. Suppose $L_{2}$ as the number of learnable layers of $M_{2}$.

Step 6: Freeze the specific early layers by setting their learning rate to 0,

(27) $$\vec{l_{r}}\left[M_{2}\left(1:L_{0}-L\right)\right]\leftarrow 0$$

where $l_{r}$ refers to the learning rate, and M(m,n) refers to the layers from *m* to *n* in the network model *M*.

Step 7: Get the new added five layers/blocks retrainable by setting their learning rate to 1,

(28) $$\vec{l_{r}}\left[M_{2}\left(L_{2}-4:L_{2}\right)\right]\leftarrow 1$$

Step 8: Import the segmented image set $T_{Seg}$.

Step 9: Retrain the whole neural network with input $T_{seg}^{train,\;i}$, Bayesian Optimization training algorithm, and get the new network $GBBNet^{i}$,

(29) $$GBBNet^{i}=F_{rn}\left(M_{2},T_{seg}^{train,\;i}\right)$$

where $F_{rn}$ refers to a function of retraining the network and $T_{seg}^{train,\;i}$ refers to the input training dataset.

Step 10: Output the test performances of GBBNet.

Finally, the pseudocode for the CSGBBNet is listed in Algorithm 2.
**Algorithm 2** Pseudocode of our proposed CSGBBNet.**Input**: Original Image set $T_{R}$ and its ground truth label $G_{t}$
**Phase I: Pre-processing** $T_{R}\to T_{Seg}$, see Algorithm 1.**Phase II: Choose the pre-trained model**Load the raw PTN model: $M_{VGG16}$ $M_{vgg19}$ $M_{GoogLeNet}$;Pre-train on them and make a comparison on their testing accuracy achieved, then choose the best one:$M_{0}$ = $Max\left[Testing\;Accuracy\left(M_{VGG16},M_{vgg19},M_{GoogLeNet}\right)\right]$;
**Phase III: Five-fold cross-validation on the Training set**Split $T_{Seg}$ into training set and testing set: $T_{Seg}\to \left\{T_{seg}^{train},\;T_{seg}^{test}\right\}$
**for** *i* = 1:5   % $T_{seg}^{train,\;i}$ is the training set, $T_{seg}^{test,\;i}$ is the testing set.  Remove the last *L* learnable layers from $M_{0}$ to get a new $M_{1}$, see Equation (25).  Add 5 new designed layers/blocks (containing Batch Normalization layers), see Equation (26).  Freeze the specific early layers, see Equation (27).  Get the new added layers retrainable, see Equation (28).  Retrain the whole neural network with input $T_{seg}^{train,\;i}$ and Bayesian Optimization training algorithm. Get a new network, see Equation (29).**end****Phase IV: Report the test performance of our proposed framework**The Training set is $T_{seg}^{train}$ and its labels $G_{t}\left(T_{seg}^{train}\right)$
The Testing set is $T_{seg}^{test}$ and its labels $G_{t}\left(T_{seg}^{test}\right)$**for** *i* = 1:5Prediction: Pred(i)= predict $(GBBNet^{i}$, $T_{seg}^{test,i}$ )Test Confusion matrix: $Conf_{Test}=\mathrm{compare}\left[Pred\left(i\right),\;G_{t}\left(T_{seg}^{test,i}\right)\right]$Calculate Indicators: accuracy, sensitivity, specificity, precision, F1 score, see Equation (30).**end****Output: The best model GBBNet and its test performances.**

**Figure 10:**
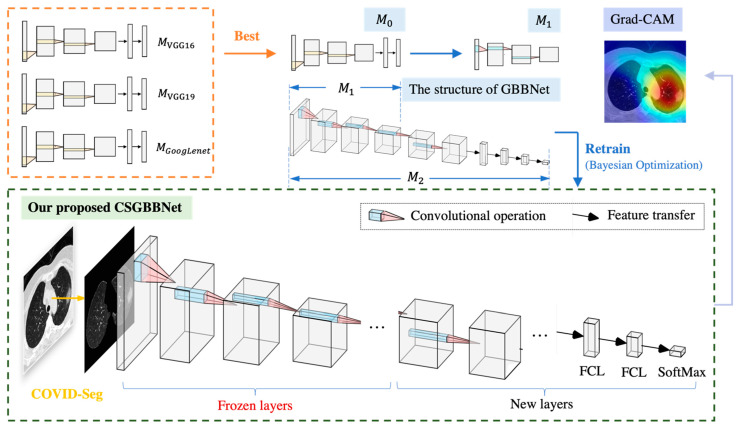
A simplified method diagram for our proposed CSGBBNet. The feature representation of each image is extracted by several convolutional operations and then be mapped into the FCL to reduce the dimension, and finally the prediction probabilities for classes are output. ($M_{0}$: The optimal selection of pre-trained models. $M_{1}$: The model achieved by removing the last L learnable layers from $M_{0}$. $M_{2}$: The model achieved by adding five new designed layers/blocks into $M_{1}$).

All in all, our experiment used a novel model COVID-Seg for image preprocessing, GBBNet for classification, and five-fold as a validation method that reports unbiased performances. We named the whole framework as CSGBBNet. Considering the factor of overfitting and running time, we intended to choose five as the *K* value for our experiment. A simplified method diagram for the running process of our proposed CSGBBNet is displayed in Figure 10. The detailed structures of ‘Frozen layers’ and ‘New layers’ are defined in Figure 6.

## 4. Experiments, Results, and Discussion

### 4.1. Data Splits

All the experiments were conducted on a laptop with GTX1060. The majority of experiments were written using MATLAB 2020a. To verify the robustness of our framework, we introduced a new benchmark dataset ‘COVID-CT’ with 539 CT scan images. In our experiment, we consider COVID-19 patient images as positive samples and healthy control images as negative samples on both datasets. Due to the reason that we utilized a fivefold cross-validation method, we would have five times of running, and in each running, images are divided into 80% for training, 20% for testing. A clear table for data splits for both the COVID Academic dataset and the new benchmark dataset can be found in Table 5.

Besides, to comprehensively evaluate the performance of the classifier, we added an evaluation criterion of harmonic average F1 score [47,48,49,50], which considers the value of both precision and recall according to the equation:

(30) $$F1\;\mathrm{score}=\frac{2\cdot \mathrm{Precision}\cdot \mathrm{Rcall}}{\mathrm{Precision}+\mathrm{Rcall}}$$

### 4.2. Statistical Results

**Figure 11:**
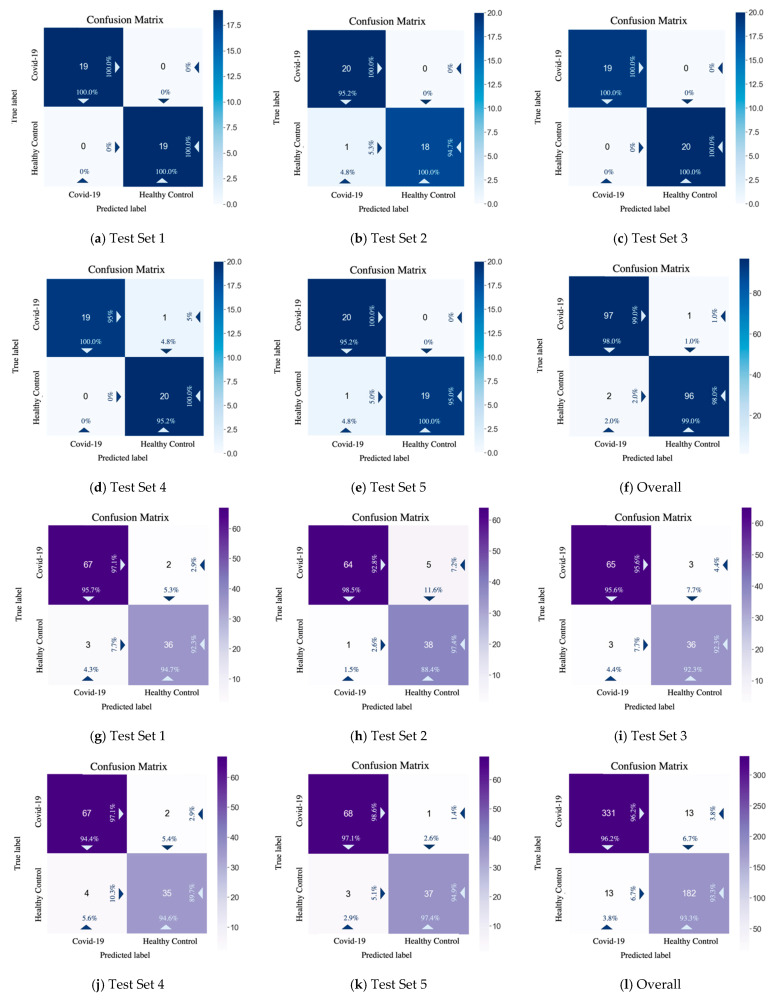
Confusion matrix results on Test Sets by CSGBBNet. (**a**–**f**): The results on the COVID Academic dataset. (**g**–**l**): The results on the COVID-CT dataset.

From the test performance and the confusion matrix results displayed in Table 6 and Figure 11, on the COVID Academic dataset, CSGBBNet predicted 193 correctly among all the 196 samples. While on the COVID-CT dataset, CSGBBNet achieves a mean accuracy of 95.17 ± 1.22%, and a mean F1 score of 96.21 ± 0.98%, which are higher than the best accuracy (89.1%) and the best F1 score (89.6%) reported in [46]. This verifies that our framework has excellent robustness when encountered different datasets.

Taking the overall confusion matrix on the COVID Academic dataset as an illustration, the classifier correctly recognized 97 images of ‘COVID-19’ as ‘COVID-19’, 96 images of ‘Healthy Control’ as ‘Healthy Control’ but misidentified two images of ‘Healthy Control’ as ‘COVID-19’ 1 image of ‘COVID-19’ as ‘Healthy Control’. In the upper-left box of the confusion matrix, the value 98.0% means 98.0% of the ‘samples’ predicted as ‘COVID-19’ were classified right and the value 99.0% means 99.0% of the real ‘COVID-19’ samples were classified right. Meanwhile, in the lower-right box, the value 99.0% means 99.0% of the ‘samples’ predicted as ‘Healthy’ were classified right, and the value 98.0% represents 98.0% of the real ‘Healthy’ samples were classified right. In a medical sense, our approach has a very low misdiagnosis rate, especially when the target is a real COVID-19 patient. This is very helpful for practical applications because the high rate of diagnosis of COVID-19 patients can effectively prevent them from the second transmission of the epidemic.

**Figure 12:**
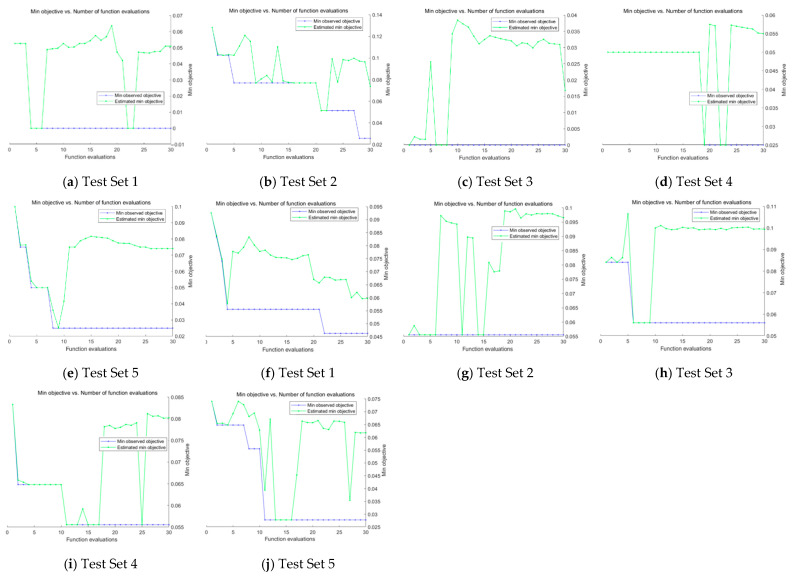
Bayesian Optimization process on Test Sets by CSGBBNet. (**a**–**e**): The results on the COVID Academic dataset. (**f**–**j**): The results on the COVID-CT dataset.

The graphs of minimum objective vs. number of function evaluations achieved during the Bayesian Optimization process on Test Sets in both datasets are shown in Figure 12, where different combinations of hyperparameter such as ‘initial learning rate’, ‘momentum’, etc. were attempted in the training process to achieve an optimum result. In the next section, we will implement an ablation study to conduct a full analysis of our results.

### 4.3. Ablation Study

To more explicit verify the effectiveness of different proposed modules in our experiment, we named the framework without utilizing the COVID-Seg model as ‘GBBNet Only’, the model only without newly added designed layers as ‘CSGBBNet no BN’, the model only without Bayesian Optimization module as ‘CSGBBNet no BO’. In this section, all the experiments were conducted on ‘COVID Academic’.

**Figure 13:**
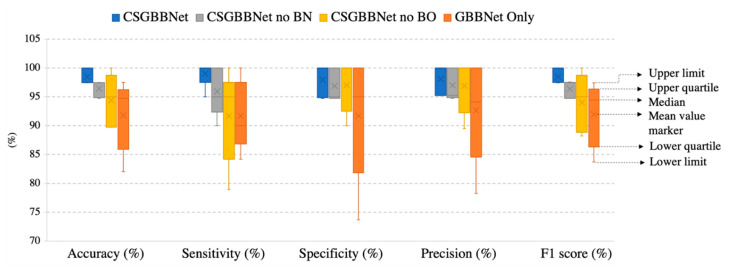
The results of our ablation study.

**Figure 14:**
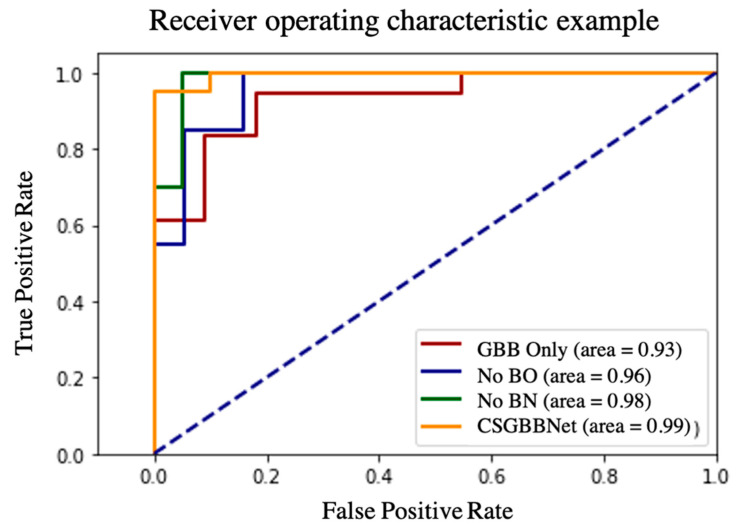
The ROC analysis curve results of our ablation study.

As displayed in Table 7 and Figure 13, firstly, after introducing the COVID-Seg model, the results in the testing set were significantly improved with the accuracy of 6.68%, the sensitivity of 7.26%, the specificity of 6.21%, the precision of 5.44%, the F1 score of 6.56%, and the standard deviation in a range of 3.36–7.25%. This means our segmentation was very successful and effective. Second, after adding newly designed layers, the accuracy in the testing set improved 2.07%, the sensitivity improved 3.05%, the specificity improved 1.06%, the precision improved 1.10%, the F1 score improved 2.13%, and the stability of results also slightly improved. Third, utilizing Bayesian Optimization can help to find the optimum hyperparameters for the model because all the evaluated criteria have different degrees of improvements: the accuracy improved with 4.11%, the sensitivity improved with 7.32%, the specificity improved with 0.95%, the precision improved with 1.21%, the F1 score improved with 4.48% and the stability improved with 0.16–5.19% revealed by standard deviation. Finally, according to the Receiver operating characteristic (ROC) curve in Figure 14, the illustrated diagnostic ability of the CSGBBNet classifier system is also superior to those without complete modules.

To sum up, the results above indicate the modules: COVID-Seg model, Bayesian Optimization training algorithm, and newly added layers are all effective in our framework.

### 4.4. Comparison between CSGBBNet and Other State-of-the-Art Approaches

We compared the proposed CSGBBNet with seven state-of-the-art approaches: WE-BO [51], GLCM-SVM [52], GoogLeNet [29], ResNet18 [53], DenseNet201 [54], VGG16 [40], VGG19 [40].

**Figure 15:**
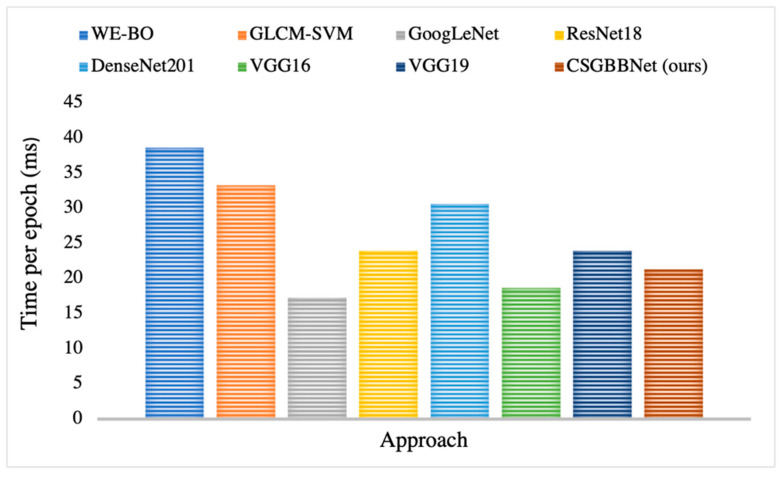
Per-epoch training time of CSGBBNet vs. other seven methods.

In Table 8, when compared with seven state-of-the-art methods on the COVID Academic dataset, CSGBBNet has advantages in terms of most of the performance metrics. The results show excellent stability according to the values provided by standard deviation. And it is clear in Figure 15 that CSGBBNet presents very good computational efficiency as it performs faster than most other approaches, except the comparative performance with GoogLeNet and VGG16. However, it can be easily observed that the speed difference among them is not significant, and our framework shows a much higher diagnostic rate when compared with GoogLeNet and VGG16.

**Figure 16:**
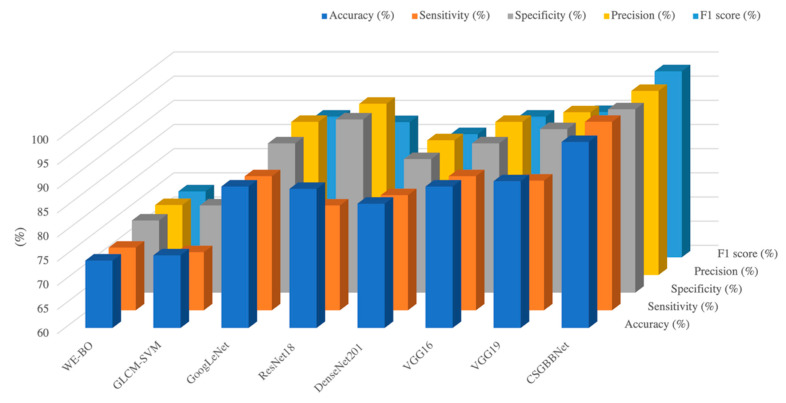
A summary of comparisons between state-of-art models and ours.

To conclude for this section, CSGBBNet achieves excellent performance with the mean accuracy of 98.49 ± 1.23%, the sensitivity of 99.00 ± 2.00%, the specificity of 97.95 ± 2.51%, the precision of 98.10 ± 2.61%, and the F1 score of 98.51 ± 1.22%. It is worth mentioning that, as illustrated in Figure 16, it performs better than seven other state-of-the-art methods in accuracy, specificity, precision, F1 score, and especially the sensitivity criterion of the model. The sensitivity of CSGBBNet is 12.64% higher than the approach ranked second in the table. The reason is we effectively removed the factors in the image that would affect the judgment of the model, such as the regions of the heart, ribs, and thoracic vertebrae. Moreover, our proposed model shows excellent stability, robustness, and very good computational efficiency when compared with other state-of-the-art machine learning approaches and the traditional RT-PCR test methods. In all, CSGBBNet has a low misdiagnosis rate, high stability, and can successfully diagnose multiple slice images within one second, which is of great significance in the practical application of COVID-19 detection.

To further verify if CSGBBNet provides an explainable and promising way to detect COVID-19, gradient-weighted class activation mapping (Grad-CAM) is employed in the next section.

### 4.5. Explainability of Proposed CSGBBNet

**Figure 17:**
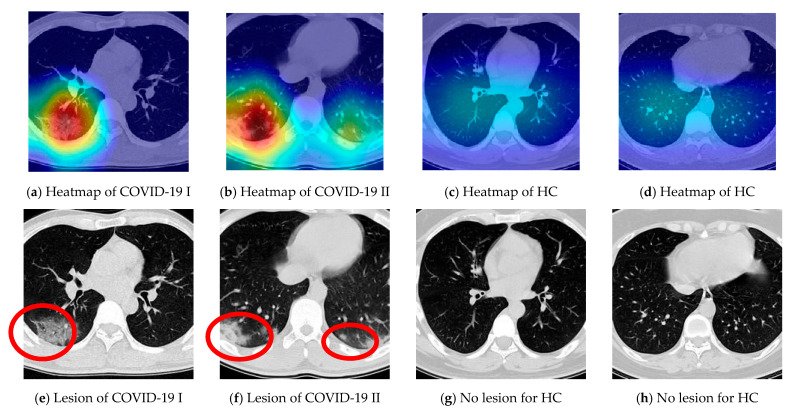
Delineation of COVID-19 samples. The lesion area in (**e**,**f**) is circled in red.

We take samples from the COVID-19 and HC class respectively and display the relevant heatmaps in Figure 17a–d to verify the explainability of CSGBBNet. The manual delineations of lesion area for them are shown in Figure 17e–h. These heatmaps were generated utilizing the ‘Inception 5b output’ feature map in GBBNet with the help of the Grad-CAM approach. The red area in the heatmap is the part with strong attention in the model, and the deep blue area is the part with little attention in the model. According to these heatmaps, we can confirm that, for the COVID-19 image (Figure 17a,b), our framework is paying more attention to the lesion areas and meanwhile paying little attention to the non-lesion areas. While for the HC image (Figure 17c,d), the attention of the model is not focused on any particular area because there is no lesion area in the healthy control category.

To sum up, the heatmaps provide a clear and understandable interpretation of how our CSGBBNet predicts COVID-19 images from healthy control images. In other words, the concerns of our model are very similar to the standard already approved in the medical community, which adds confidence that it is capable of assisting the diagnosis of doctors, radiologists, and inspectors at each epidemic prevention site in the real world.

## 5. Conclusions

Chest CT is a rapid, non-invasive method for screening COVID-19. Our proposed deep learning framework CSGBBNet using the COVID-Seg model as an image preprocessing method, GBBNet as a classification method is also an explainable framework to accomplish the classification task. CSGBBNet not only shows advantages in diagnostic rate, stability, and computational efficiency when compared with other state-of-the-art machine learning approaches and traditional RT-PCR tests, but also shares similar concerns with the standard approved in the medical community. Moreover, it shows excellent robustness when encountered with different datasets. To conclude, combining these two techniques can help to realize rapid, effective, stable, and safe detection of COVID-19, which is of great significance to clinical medicine and society.

In the future, we plan to incorporate or learn from more advanced deep learning techniques and revise the model to improve classification efficiency.

## Figures and Tables

**Table 1:** A survey of latest proposed deep learning techniques in detecting COVID-19.

Reference	Number of Scans in the Dataset	Method(s)	Reported Accuracy
Wang et al. [13]	COVID-19 (358) Non-COVID19 Pneumonia (5538) Normal (8066)	**COVID-Net**	**93.3%**
		ResNet-50	90.6%
		VGG-19	83%
Dey et al. [14]	COVID-19 (200) Normal (200)	**Feature fusion + KNN**	**87.75%**
		Feature fusion + SVM-RBF	87.25%
		Feature fusion + RF	86.75%
		Feature fusion + DT	86.50%
Abbas et al. [15]	COVID-19 (105) SARS (11) Normal (80)	**DeTraC + ResNet**	**93.10%**
		**DeTraC + VGG19**	**93.10%**
		DeTraC + GoogLeNet	89.65%
		DeTraC + AlexNet	89.10%
		DeTraC + SqueezeNet	82.75%

(The best methods and results on each dataset are shown in bold).

**Table 2:** Abbreviation list.

Abbreviation	Representation
FCL	fully connected layer
BN	batch normalization
AC	acquisition function
UCB	upper confidence bound
PI	probability of improvement
EI	expected improvement
CDF	cumulative distribution function
PTN	pre-trained network

**Table 3:** Symbol list.

Symbol	Meaning
$F_{resize}$	function of resizing the images to a same size
$F_{mets}$	function of utilizing Maximum entropy threshold segmentation-based method
$F_{ed}$	function of ‘erosion and dilation-based process’
$F_{crwc}$	function of choosing and retaining the wanted connectivity areas
$F_{comb}$	function of conducting element-wise multiplication process between the final refined mask and the raw image to get the final segmented image
$F_{rl}$	function of removing layers
$F_{andl}$	function of adding newly designed layers
$l_{r}$	learning rate
$F_{rn}$	function of retraining the network

**Table 4:** Bayesian Optimized Variable Setting.

Name of Optimized Hyperparameter	Range
InitialLearnRate	(1 × 10^−5^, 1)
Momentum	(0.8, 0.98)
L2Regularization	(1 × 10^−10^, 1 × 10^−2^)
LearnRateDropFactor	(0, 1)

**Table 5:** Data Splits.

Dataset	Label	Range of Number of Training Frames	Range of Number of Testing Frames	Overall
COVID Academic	COVID-19 (positive)	78–79	19–20	98
	Healthy (negative)	78–79	19–20	98
	Overall	156–158	38–40	196
COVID-CT	COVID-19 (positive)	280–281	68–69	349
	Healthy (negative)	151–152	38–39	190
	Overall	431–433	106–108	539

(‘COVID Academic’ is referenced from [18]. ‘COVID-CT’ is referenced from [46]).

**Table 6:** Test performance based on CSGBBNet.

Dataset	Test Set	Accuracy (%)	Sensitivity (%)	Specificity (%)	Precision (%)	F1 Score (%)
COVID Academic	1	100.00	100.00	100.00	100.00	100.00
	2	97.44	100.00	94.74	95.24	97.56
	3	100.00	100.00	100.00	100.00	100.00
	4	97.50	95.00	100.00	100.00	97.44
	5	97.50	100.00	95.00	95.24	97.56
	Mean + SD	98.49 ± 1.23	99.00 ± 2.00	97.95 ± 2.51	98.10 ± 2.61	98.51 ± 1.22
COVID-CT	1	95.37	97.10	92.31	95.71	96.40
	2	94.44	92.75	97.44	98.46	95.52
	3	94.39	95.59	92.31	95.59	95.59
	4	94.44	97.10	89.74	94.37	95.71
	5	97.22	98.55	94.87	97.14	97.84
	Mean + SD	95.17 ± 1.22	96.22 ± 2.20	93.33 ± 2.92	96.25 ± 1.58	96.21 ± 0.98

(SD = standard deviation).

**Table 7:** Results of our ablation study.

Approach	Accuracy (%)	Sensitivity (%)	Specificity (%)	Precision (%)	F1 Score (%)
GBBNet Only	91.81 ± 5.49	91.74 ± 5.36	91.74 ± 9.76	92.66 ± 8.00	91.95 ± 4.99
CSGBBNet no BN	96.42 ± 1.27	95.95 ± 3.76	96.89 ± 2.54	97.00 ± 2.46	96.38 ± 1.34
CSGBBNet no BO	94.38 ± 1.39	91.68 ± 7.19	97.00 ± 4.00	96.89 ± 4.19	94.03 ± 4.53
**CSGBBNet**	**98.49** ± **1.23**	**99.00** ± **2.00**	**97.95** ± **2.51**	**98.10** ± **2.61**	**98.51** ± **1.22**

(Our methods and results are shown in bold).

**Table 8:** Comparison with state-of-the-art approaches on the test set.

Approach	Accuracy (%)	Sensitivity (%)	Specificity (%)	Precision (%)	F1 Score (%)	Per-Epoch Training Time (ms)
WE-BO [51]	73.95 ± 0.98	72.97 ± 2.96	74.93 ± 2.39	74.48 ± 1.34	73.66 ± 1.33	38.67
GLCM-SVM [52]	75.03 ± 1.12	72.03 ± 2.94	78.04 ± 1.72	76.66 ± 1.07	74.24 ± 1.57	33.33
GoogLeNet [29]	88.27 ± 2.04	83.63 ± 3.95	92.84 ± 5.15	92.61 ± 4.81	87.72 ± 1.98	17.33
ResNet18 [53]	88.78 ± 2.58	81.68 ± 2.06	95.84 ± 3.93	95.43 ± 4.19	87.98 ± 2.45	24.00
DenseNet201 [54]	89.24 ± 6.58	82.58 ± 9.12	95.89 ± 5.03	95.11 ± 6.03	88.29 ± 7.46	30.67
VGG16 [40]	89.28 ± 1.95	87.74 ± 6.83	90.89 ± 9.18	91.67 ± 7.58	89.13 ± 1.72	18.67
VGG19 [40]	90.37 ± 5.12	86.84 ± 4.96	93.84 ± 6.08	93.67 ± 5.98	90.08 ± 5.13	24.00
**CSGBBNet (ours)**	**98.49** ± **1.23**	**99.00** ± **2.00**	**97.95** ± **2.51**	**98.10** ± **2.61**	**98.51** ± **1.22**	**21.33**

(Our methods and results are shown in bold).
